# New Morphological and Molecular Data on *Lobatostoma anisotremum* Oliva & Carvajal, 1984 (Trematoda: Aspidogastridae) from *Anisotremus scapularis* (Tschudi, 1846) (Eupercaria *incertae sedis*: Haemulidae) off the Peruvian Coast, with Insights into the Phylogeny and Systematics of *Lobatostoma*

**DOI:** 10.3390/ani16121873

**Published:** 2026-06-17

**Authors:** Alex Oscco, Nicolas Tarmeño, Celso L. Cruces, Jefferson Yunis-Aguinaga, Jhon D. Chero

**Affiliations:** 1 Laboratorio de Zoología de Invertebrados, Departamento Académico de Zoología, Facultad de Ciencias Biológicas (FCB), Universidad Nacional Mayor de San Marcos (UNMSM), Av. Universitaria Cruce con Av. Venezuela Cuadra 34, Lima 15088, Peru; alexoscco97@gmail.com (A.O.); ntarmenor@unmsm.edu.pe (N.T.); jcherod@unmsm.edu.pe (J.D.C.); 2 Facultad de Medicina Humana, Universidad Ricardo Palma, Lima 15039, Peru; celso.cruces@urp.edu.pe; 3 Facultad de Ciencias Veterinarias y Biológicas, Universidad Científica del Sur, Carretera Panamericana Sur, Km 19, Lima 150142, Peru

**Keywords:** Aspidogastrea, parasite diversity, integrative taxonomy, phylogenetic inference

## Abstract

The aspidogastrean genus *Lobatostoma* Eckmann, 1932 remains poorly understood, with limited morphological and molecular data hindering its systematics and phylogenetic resolution. In the present study, we provide the first integrative characterisation of *Lobatostoma anisotremum* Oliva & Carvajal, 1984 from the southeastern Pacific coast of Peru, combining detailed morphological, ultrastructural and molecular analyses. A total of 137 specimens were recovered from the intestine of 65 Peruvian grunt, *Anisotremus scapularis* (Tschudi, 1846) (Haemulidae), with a prevalence of 43% and mean abundance of 2.1 ± 3.4 parasites per host. Morphological observations based on light microscopy and scanning electron microscopy (SEM) allowed the refinement of diagnostic features, including tegumental papillae distribution, marginal organs, and reproductive structures. Molecular characterisation included the generation of novel sequences for the 18S rDNA and 28S rDNA gene fragments, representing the first comprehensive molecular dataset for *L*. *anisotremum*. Phylogenetic analyses based on 18S and 28S rDNA datasets consistently recovered *L*. *anisotremum* as sister to *L*. *manteri* Rohde, 1973, with strong support in the 28S analysis. In contrast, *L*. *kemostoma* (MacCallum & MacCallum, 1913) was recovered as a distinct and independent lineage, clearly separated from other congeners, supporting previous hypotheses of non-monophyly within *Lobatostoma*. These findings could be further supported by consistent morphological differences, particularly in cephalic lobe configuration, hindbody length and number of alveoli on the ventral disc. Our results also suggest that current family- and subfamily level classifications within Aspidogastridae Looss, 1901 may not reflect evolutionary relationships, as evidenced by the nested position of Multicalycidae Gibson & Chinabut, 1984 and the apparent non-monophyly of traditionally defined subfamilies. Although our data supports the distinctiveness of certain lineages, including *L*. *kemostoma*, we refrain from proposing formal taxonomic changes due to limited taxon sampling and the need for a comprehensive revision of the group. Overall, this study highlights the importance of integrative approaches in resolving the systematics of Aspidogastrea Faust & Tang, 1936 and provides a robust framework for future taxonomic and phylogenetic studies.

## Simple Summary

Parasites are a natural part of any ecosystems, but many species are still poorly known, especially along the Pacific coast of South America. We investigated a parasitic flatworm that lives in the intestine of a common coastal fish from Peru. Our aim was to better understand its body structure and its evolutionary relationships with similar parasites. We used detailed imaging techniques to observe its external features and analysed its genetic information to compare it with related species. Our results confirmed the identity of the parasite and revealed new structural details that had not been described before. We also found that some closely related species may not belong to the same evolutionary group as previously believed. These findings help improve the classification of these parasites and provide new information about their diversity and evolution. This research is important because understanding parasite diversity can support better management of marine resources and contribute to a more complete view of biodiversity in marine ecosystems.

## 1. Introduction

The class Trematoda Rudolphi, 1808 (Platyhelminthes: Neodermata) includes the subclass Aspidogastrea Faust & Tang, 1936 [1,2], the least diverse group within trematodes, comprising approximately 80 described species with a worldwide distribution [3,4]. Aspidogastreans lack significant economic or medical importance but are of considerable scientific interest due to their distinctive morphology, mainly the presence of a ventral holdfast organ, their predominantly monoxenous life cycle involving molluscs as obligate host and vertebrates (fishes and turtles) as facultative or obligate definitive host in marine and freshwater environments [5], and their basal phylogenetic position within trematodes [4,6,7]. Despite this, their systematics remains poorly resolved, largely due to limited sampling and restricted availability of material, resulting in persistent uncertainties in both taxonomy and phylogeny [8,9,10,11,12].

Members of the genus *Lobatostoma* Eckmann, 1932, the most speciose genera within Aspidogastrea, are defined by the following combination of morphological characters: (1) a ventral adhesive disc bearing four longitudinal rows of alveoli; (2) the presence of cephalic lobes, including 3 ventrales and 2 dorsal lobes; (3) a single testis; and (4) a well-developed cirrus-sac [4,13]. Currently, nine species are recognised within the genus, namely, *L*. *anisotremum* Oliva & Carvajal, 1984; *L*. *jungwirthi* Kritscher, 1974; *L*. *hanumanthai* Narasimhulu & Madhavi, 1980; *L*. *veranoi* Oliva & Luque, 1989; *L*. *kemostoma* (MacCallum & MacCallum, 1913); *L*. *ringens* (Linton, 1905) (type-species); *L*. *manteri* Rohde, 1973; *L*. *platense* Mañé-Garzón & Holcman-Spector, 1976; and *L*. *pacificum* Manter, 1940 [3,14]. Six of these species have been described primarily from carangid fish, where the remaining species have been described from haemulid and sciaenid hosts, as well as from a freshwater cichlid host in the case of *L*. *jungwirthi* [3,14].

The aspidogastrean *L*. *anisotremum* was originally described from the intestine of *Anisotremus scapularis* (Tschudi, 1846) (Haemulidae) in Chile [15]. The original description, based on light microscopy and scanning electron microscopy (SEM), provided a general account of its morphology but lacked detailed information on several diagnostic structures observable under SEM, including the number and distribution of tegumental papillae, the shape and number of marginal organs, genital pore ornamentation, the position of Laurer’s canal and cirrus morphology. These features have subsequently been documented in other species of *Lobatostoma* and may represent informative characters for generic or species-level delimitation [12,16]. Since its original description, studies on *L*. *anisotremum* have primarily focused on its geographical distribution [17] and ecological parameters [18,19], whereas detailed morphological and ultrastructural studies have remained scarce.

Molecular data provides an essential complement to morphological approaches in trematode systematics [20,21,22]. In Aspidogastrea, both nuclear (18S, 28S and ITS rDNA) and mitochondrial (COI) markers have been employed for species delimitation and phylogenetic inference [23,24,25]. However, genetic data within *Lobatostoma* remains scarce and fragmentary, being available for only three species. In the case of *L*. *anisotremum*, only a short partial 18S rDNA sequence (414 bp) is currently available. Moreover, comparable data for this marker across other aspidogastreans are limited, restricting its utility for robust phylogenetic inference. Therefore, the incorporation of additional molecular markers is necessary to improve taxonomic resolution and to better resolve the phylogenetic position of *L*. *anisotremum* within Aspidogastrea.

The aim of the present study was to provide new morphological insights based on SEM and to generate novel molecular sequences (18S and 28S) for *L*. *anisotremum* to clarify its phylogenetic position within Aspidogastrea.

## 2. Materials and Methods

### 2.1. Collection of Samples and Morphological Study

Between March and September 2025, a total of 65 specimens of *A*. *scapularis* were captured off El Frontón Island (12°06′ S, 77°10′ W), Lima, Central Peru. Hosts were subjected to systematic necropsy for the detection and recovery of helminths. Trematodes were collected from the intestine, rinsed in 0.85% saline solution and fixed in hot 70% ethanol. Specimens intended for light microscopy were stained with iron acetocarmine, dehydrated through a graded ethanol series, cleared in methyl salicylate and mounted in Canada balsam. Observation and photomicrographs were obtained using a Nikon™ Eclipse Ni-L compound microscope (Nikon, Tokyo, Japan) equipped with normal bright-field and differential interference contrast microscopy (DIC). Measurements were taken in micrometres (µm), unless otherwise stated, as straight-line distances between the extreme points of the structures and are presented as the range followed by the mean and the number of structures measured (n) in parentheses. For scanning electron microscopy (SEM), selected specimens were post-fixed in 10% formalin, dehydrated through a graded ethanol series, subjected to critical point drying using carbon dioxide, sputter-coated with gold, and examined using an Inspect S50 (FEI Company, Brno, Moravia, Czech Republic), at an accelerating voltage of 5–10 kV. Specimens were deposited in the Helminthological Collection of the Museum of Natural History at the San Marcos University (MUSM-HEL), Lima, Peru.

### 2.2. Molecular Analysis

Genomic DNA of three specimens was extracted using an gSYNC™ DNA Extraction Kit (Genaid Biotech Ltd., New Taipei City, Taiwan) following the manufacturer’s protocol. A partial fragment of the 28S rDNA gene was amplified by polymerase chain reactions (PCR) using the primers 28F (5′–GTCC GATA GCGA ACAA GTAC CGT–3′) and 28R (5′–AGCA TAGT TCAC CATC TTTC GGGT CTCAA–3′) [26]. A partial fragment of the 18S rDNA gene was amplified using the primers 18F (5′–TACC TGGT TGAT CCTG CCAG TAG–3′) and 18R (5′–GATC CTTC CGCA GGTT CACC TAC–3′) [27]. PCR assays were carried out in a total volume of 25 µL containing 12.5 µL of PCRBIO Taq Mix Red (PCR Biosystems, London, UK), 0.3 µL of each primers with final concentration at 10 µM, 1.2 µL of DNA sample and ultrapure water to complete. An annealing temperature of 53 °C was used for amplification of 28S rDNA and 56 °C for 18S rDNA genes. PCR products were purified with the kit GenepHlow Gel/PCR Kit (Geneaid, Taiwan) following the manufacturer’s instructions and sequenced in Sangon Biotech (Shanghai, China) with the Sanger sequencing method.

### 2.3. Phylogenetic Analyses of Molecular Data

Sequences were edited and contig was assembled using Geneious 2026.0.2. Similar sequences were retrieved from the National Center for Biotechnology Information (NCBI) database (henceforth ‘GenBank’) using the Basic Local Alignment Search Tool (BLAST) (2.16.0). The 28S rDNA and 18S rDNA sequences generated in the present study were aligned with reference sequences representing recognised species and putative lineages of Aspidogastrea available in GenBank (see Table 1).

Multiple sequence alignments were performed using the algorithm Muscle in MEGA 12 [38]. For the phylogenetic analyses, sequences of *Polystomoides siebenrockiellae* (Rohde, 1965) and *Polystomoides oris* Paul, 1938 were used as outgroup taxa for the 28S and 18S datasets, respectively (GenBank accession numbers provided in Table 1). Bayesian phylogenetic analyses were conducted using MrBayes v3.2 [39] implemented in Geneious. The best-fit nucleotide substitution models for each dataset were selected under the Bayesian Information Criterion (BIC) using jModelTest [40]. For the 18S rDNA dataset, the K80+G model was selected. This model was implemented in MrBayes by specifying two substitution rate categories (nst = 2) and gamma-distributed rate variation among sites (rates = gamma), assuming equal base frequencies (statefreqpr = fixed(equal)). For the 28S rDNA dataset, the HKY+I+G model was selected. Accordingly, analyses were performed using nst = 2 with both a proportion of invariable sites and gamma-distributed rate variation (rates = invgamma) and allowing unequal base frequencies (statefreqpr = dirichlet (1,1,1,1)). For both datasets, two independent runs with four Markov chains each were executed for 1,000,000 generations, sampling every 1000 generations. Convergence diagnostics were evaluated using the average standard deviation of split frequencies (<0.01) and ESS values (>200). The first 25% of sampled trees were discarded as burn-in, and the remaining trees were used to construct a majority-rule consensus tree with posterior probability support values.

## 3. Results

A total of 137 specimens of *L*. *anisotremum* were recovered from the intestine of *A*. *scapularis*. Of the 65 hosts examined, 28 were infected (43%). The mean intensity (MI) was 4.9 ± 3.7 parasites per infected host (1–15), and the mean abundance (MA) was 2.1 ± 3.4 parasites per host.

Trematoda Rudolphi, 1808
Aspidogastrea Faust & Tang, 1936
Aspidogastridae Looss, 1901
*Lobatostoma* Eckmann, 1932
*Lobatostoma anisotremum* Oliva & Carvajal, 1984
Figure 1 and Figure 2A–H

**Figure 1:**
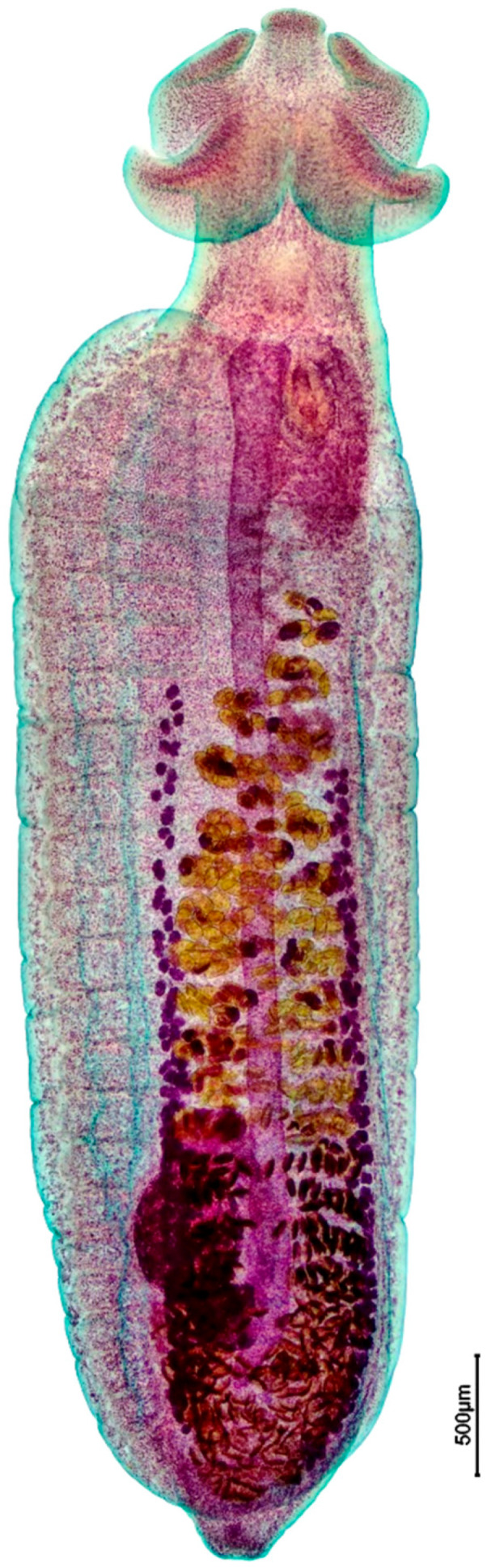
Photomicrograph of *Lobatostoma anisotremum* Oliva & Carvajal, 1984 from *Anisotremus scapularis* (Tschudi, 1845) (Eupercaria *incertae sedis*: Haemulidae) off the coast of Peru.

**Figure 2:**
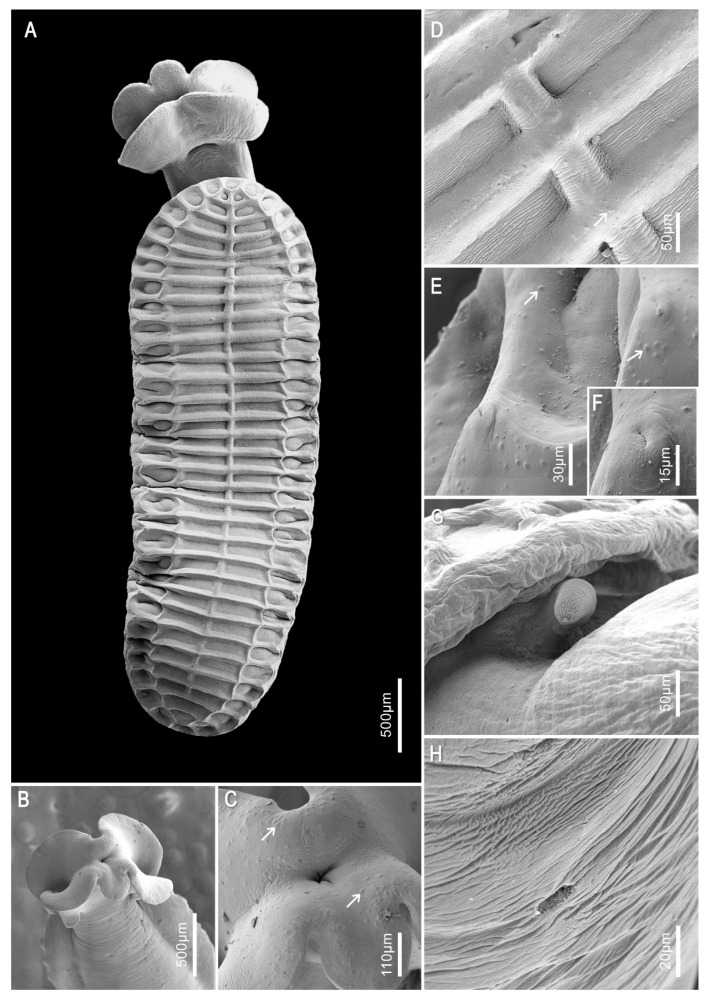
Scanning Electron Microscope (SEM) of *Lobatostoma anisotremum* Oliva & Carvajal, 1984 from *Anisotremus scapularis* (Tschudi, 1845) (Eupercaria *incertae sedis*: Haemulidae) off the coast of Peru. (**A**) Whole mount. (**B**) Anterior end. (**C**) Mouth with papillae on either side (arrow). (**D**) Papillae on the transverse and longitudinal septa (arrow). (**E**) Papillae on the marginal alveoli (arrows). (**F**) Marginal organ. (**G**) Cirrus. (**H**) Pore of the Laurer’s canal.

Supplemental data (based on 12 specimens stained with iron acetocarmine and 3 specimens prepared for SEM). Body elongate, fusiform (Figure 1 and Figure 2A), 5.63–9.73 (7.10; n = 11) mm long and 0.86–2.07 (1.38; n = 11) mm wide at the mid-level of the ventral disc. Tegument unarmed (Figure 2A). Ventral disc oval, with three longitudinal and 56–66 transverse septa, bearing inconspicuous papillae irregularly distributed (Figure 2A), 4.49–8.25 (5.51; n = 12) mm long and 1.11–2.42 (1.77; n = 12) mm wide, occupying 70–85% (77%; n = 12) of body length.

Marginal organs 56–66 (61; n = 11) in number (Figure 2A), 32–74 (50; n = 9) in diameter, arranged along the margin of the ventral disc (Figure 2A,E). Alveoli 110–130 (118; n = 11) in total, comprising central and marginal types; marginal alveoli 56–66 (61; n = 11) in number (Figure 2A), 105–200 (150; n = 12) long and 150–410 (280; n = 12) wide; central alveoli 27–32 (30; n = 11) in number (Figure 2A), 80–160 (125; n = 12) long and 210–510 (388; n = 12) wide. Mouth surrounded by well-developed lips (two ventral and three dorsal), with small papillae irregularly distributed (Figure 2C). Forebody 1.05–1.99 (1.39; n = 12) mm long. Hindbody tubular, short, 110–700 (350; n = 11) long, representing approximately 7.2% of total body length (Figure 1). Prepharynx long, straight, 0.79–1.40 (1.00; n = 12) mm long. Distance from genital pore to anterior extremity 1.08–2.47 (1.49; n = 12) mm. Pharynx elongate, muscular-glandular, 230–400 (280; n = 12) long and 210–390 (270; n = 12) wide (Figure 2B). Intestinal caecum single, sac-like, extending posteriorly to the level of the testis (Figure 1). Testis single, subspherical, slightly dextral, 0.42–1.24 (0.76; n = 12) mm long and 0.39–1.08 (0.60; n = 12) mm wide, located in the posterior third of the body (Figure 1). Seminal vesicle 80–240 (140; n = 12) long, situated at the mid-body level (Figure 1). Cirrus sac pyriform, 330–830 (580; n = 12) long. Cirrus 130–640 (310; n = 12) long, muscular, tapered (Figure 2G). Pharynx to cirrus sac length ratio 1:1.5–1:2.4 (1:2; n = 12). Ovary oval, pretesticular, 270–790 (460; n = 12) long and 210–440 (300; n = 12) wide. Metraterm 0.62–1.26 (0.85; n = 11) mm long. Vitelline glands follicular, extending from the mid-disc region to the posterior third of the body at the level of the testis (Figure 1). Laurer’s canal opening dorsal, located in the posterior third of the body (Figure 2H). Eggs oval (Figure 1), 85–119 (104; n = 12) long and 45–61 (53; n = 12) wide.

Host: *Anisotremus scapularis* (Tschudi, 1845) (Eupercaria *incertae sedis*: Haemulidae), Peruvian grunt.

Locality: off El Frontón Island (12°06′ S, 77°10′ W), Lima, Central Peru.

Site in host: Intestine.

Deposited specimens: 4 voucher specimens (MUSM-HEL 5670a-d).

Representative DNA sequences: PZ297966 (18S rDNA) and PZ297967 (28S rDNA).

### Molecular Analysis

**Figure 3:**
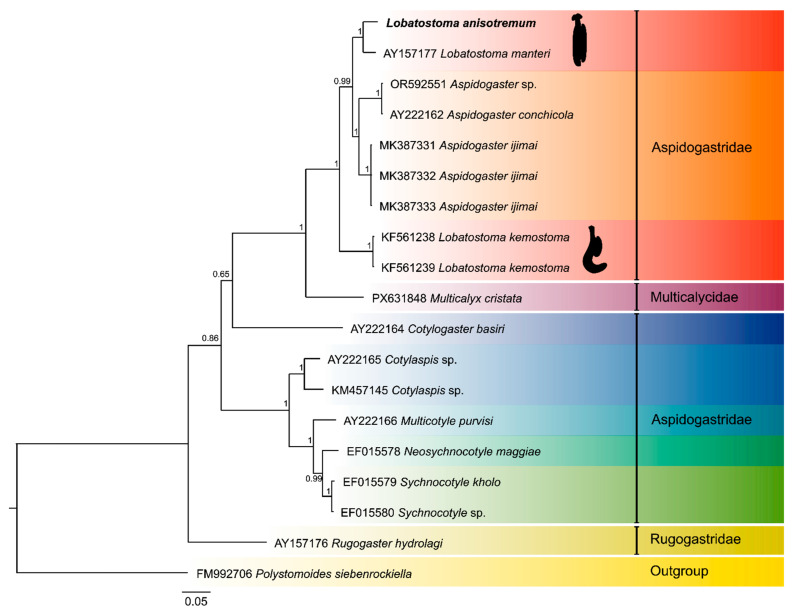
Phylogenetic tree of species/lineages of Aspidogastrea through the 28S rDNA gene inferred using Bayesian Inference (BI). Sequence obtained in this study is in bold. Scale-bar: average number of nucleotide substitutions per site.

**Figure 4:**
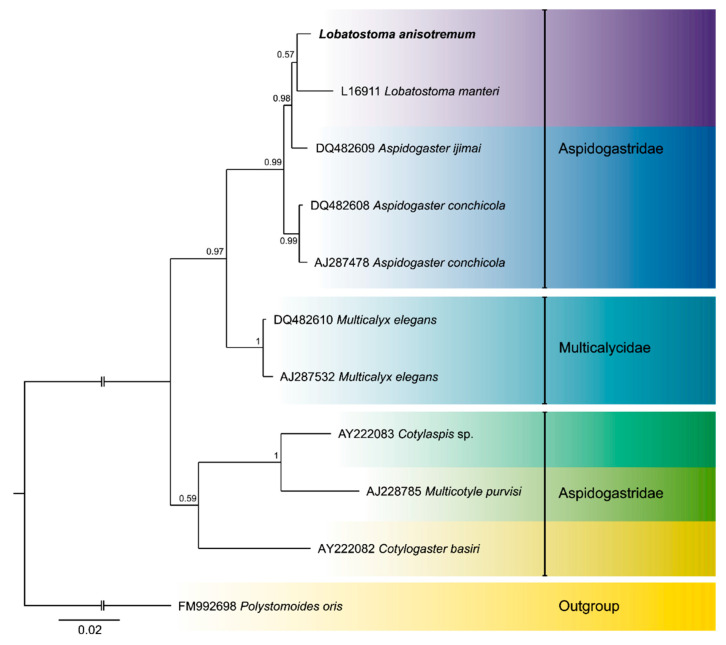
Phylogenetic tree of species/lineages of Aspidogastrea through the 18S rDNA gene inferred using Bayesian Inference (BI). Sequence obtained in this study is in bold. Scale-bar: average number of nucleotide substitutions per site.

Sequences of two ribosomal markers were obtained, including a nearly complete sequence of the 18S rDNA (1892 bp) and a partial 28S rDNA sequence (903 bp). The 18S rDNA alignment included 11 taxa and 2000 characters, of which 1534 were conserved, 397 were variable, and 158 were parsimony-informative. The 28S rDNA alignment comprised 19 taxa and 1329 characters, of which 650 were conserved, 600 were variable, and 419 were parsimony-informative. Bayesian inference analyses of the 28S dataset yielded a mean estimated marginal likelihood of −7178.2 (median = −7177.71), with effective sample size (ESS) values for all parameters up to 200. For the 18S dataset, the mean estimated marginal likelihood was −5576.795 (median = −5576.446), and ESS values also indicated adequate mixing and convergence (ESS > 700). The trees inferred under the Bayesian inference (BI) framework showed congruent topologies for both nuclear markers (18S and 28S) (Figure 3 and Figure 4).

The phylogenetic reconstruction based on the 28S rDNA dataset grouped *L*. *anisotremum* with *L*. *manteri* (AY157177) in a fully supported clade (BI = 1.00). This lineage was recovered as sister to a clade comprising *Aspidogaster* sp. (OR592551), *A*. *conchicola* (AY222162) and *A*. *ijimai* (MK387331–33), with strong support (BI = 0.99). *Lobatostoma kemostoma* (KF561238–39) was recovered as an independent lineage (BI = 1.00), positioned outside the clade formed by *L*. *anisotremum*, *L*. *manteri* and *Aspidogaster* spp., suggesting the non-monophyly of *Lobatostoma*. *Multicalyx cristata* Faust & Tang, 1936 (PX631848) (Multicalycidae) was nested within Aspidogastridae and recovered as sister to the clade comprising *Lobatostoma* spp. and *Aspidogaster* spp., with strong support. *Rugogaster hydrolagi* Schell, 1973 (AY157176) (Rugogastridae) occupied a basal position within Aspidogastrea.

The phylogenetic reconstruction based on the 18S rDNA dataset grouped *L*. *anisotremum* with *L*. *manteri* (L16911), forming a not supported clade (BI = 0.57). This clade was recovered as sister to *A. ijimai* (DQ482609), with strong support (BI = 0.98). The resulting assemblage (*Lobatostoma* spp. + *A*. *ijimai*) was in turn recovered as sister to *A*. *conchicola* (DQ482608, AJ287478), with strong support (BI = 0.99). *Multicalyx elegans* (DQ482610, AJ287532), as observed in the 28S rDNA phylogeny, was nested within Aspidogastridae and recovered as sister to the clade comprising *Lobatostoma* spp. and *Aspidogaster* spp. (BI = 0.97). This larger clade was, in turn, recovered as sister to a basal assemblage within Aspidogastridae comprising *Cotylaspis* sp. (AY222083), *Multicotyle purvisi* (AJ228785) and *Cotylogaster basiri* (AY222082).

## 4. Discussion

The present study represents the first integrative characterisation of *L*. *anisotremum* in South America, combining morphological, ultrastructural and molecular data to confirm its specific identity and provide new insights into its phylogenetic position within Aspidogastrea. In addition, this study provides the first 28S rDNA sequences for *L*. *anisotremum*, thereby significantly improving the molecular representation of the genus.

Phylogenetic analyses based on nuclear markers (18S and 28S rDNA) recovered a consistent topology supporting a close relationship between *L*. *anisotremum* and *L. manteri*. This association was strongly supported in the 28S dataset and moderately supported in the 18S analysis, a pattern likely reflecting the slower evolutionary rate and limited resolution of the 18S marker at lower taxonomic levels. The clustering of these two species may be further supported by shared morphological and biogeographical traits. Both species exhibit a relatively short hindbody and a similar configuration of the cephalic region, particularly in having three dorsal lobes of comparable size [3]. In addition, both species are distributed in the Pacific Ocean, although their known distributions are widely separated, with *L*. *anisotremum* occurring in *A*. *scapularis* from the southeastern Pacific off the coast of Peru and Chile, whereas *L*. *manteri* has been reported from *Trachinotus blochii* (Lacepède, 1801) in the western Pacific off Australia.

In our 28S rDNA analysis, *L*. *kemostoma*, an Atlantic species parasitizing *Trachinotus carolinus* (Linnaeus, 1766) from Brazil [41], was recovered as a distinct and independent lineage, clearly separated from the clade comprising *L. anisotremum* and *L. manteri*. This result provides additional support for the non-monophyletic status of *Lobatostoma*, as previously suggested by Alves et al. [3] and Sokolov et al. [25]. The genus *Lobatostoma* was established by Eckmann [41] to accommodate two species originally described under *Aspidogaster* von Baer, 1826, *Aspidogaster ringens* Linton, 1905 and *Aspidogaster kemostoma* MacCallum & MacCallum, 1913. Eckmann [41] defined the genus based on the following combination of characters, i.e., a ventral adhesive disc with four rows of alveoli, marginal organs restricted to the edge of the disc, a mouth opening surrounded by lobular projections, and the presence of a single testis. However, the current diagnosis of the genus appears to group species that do not share a common evolutionary origin. The distinct phylogenetic position of *L. kemostoma* suggests that this species may represent a separate evolutionary lineage within Aspidogastrea, potentially warranting recognition at the generic level. This hypothesis could be further supported by consistent morphological differences between *L*. *kemostoma* and the clade formed by *L*. *anisotremum* and *L*. *manteri*. *Lobatostoma kemostoma* exhibits dorsal cephalic lobes of unequal shape and size (with the central lobe smaller than lateral ones) and a relatively long hindbody (tail), representing approximately half of the total body length [3]. In contrast, *L*. *anisotremum* and *L*. *manteri* possess dorsal cephalic lobes of equal shape and size and a shorter hindbody, comprising less than one-third of the total body length. Additionally, *L*. *anisotremum* and *L*. *manteri* have more than 100 alveoli on the ventral disc, whereas *L*. *kemostoma* has fewer than 70 [15,16]. Together, these differences could further support the distinctiveness of *L*. *kemostoma* within the genus. However, given the limited taxon sampling currently available for *Lobatostoma* spp., as well as the lack of a comprehensive morphological reassessment of all described species, we refrain from proposing taxonomic changes at this stage. A robust revision of the genus will require broader sampling, including additional species and molecular markers, as well as a critical re-evaluation of diagnostic morphological characters to identify potential synapomorphies.

Although molecular-based systematic studies on Aspidogastrea remain limited, four families are currently recognised within two orders [14]. The order Stichocotylida Gibson & Chinabut, 1984 includes the Stichocotylidae Faust & Tang, 1936, whereas the order Aspidogastrida Dollfus, 1958 comprises the Aspidogastridae Poche, 1907; Multicalycidae Gibson & Chinabut, 1984; and Rugogastridae Schell, 1973 [14]. However, in our phylogenetic analyses based on both the 28S and 18S datasets, the Multicalycidae, represented by *Multicalyx cristata* (Faust & Tang, 1936) in the 28S tree and *M*. *elegans* in the 18S tree, was consistently recovered as nested within a major clade comprising representatives of Aspidogastridae, thereby rendering Aspidogastridae non-monophyletic. Morphologically, Multicalycidae, currently comprising only two species, has been characterised by the presence of a single caecum and a ventral disc bearing a single longitudinal row of alveoli separated by transverse septa [5]. The Aspidogastridae, the most diverse family within Aspidogastrea, are defined by the presence of a single caecum and a ventral disc typically bearing three or four longitudinal rows of alveoli [5]. Despite these differences, both Multicalycidae and Aspidogastridae share key morphological features, including the presence of a single caecum and a ventral disc with alveoli arranged in longitudinal rows, albeit with variation in the number of rows. Considering these findings, our results suggest that the recognition of Multicalycidae as a distinct family may not be phylogenetically justified, and that its representatives could be more appropriately accommodated within Aspidogastridae. Nevertheless, broader taxon sampling and additional molecular data will be necessary before formal taxonomic changes can be confidently proposed.

Aspidogastridae currently comprises three subfamilies: Rohdellinae Gibson & Chinabut, 1984, Cotylaspidinae Chauhan, 1954 and Aspidogastrinae Poche, 1907. Members of Cotylaspidinae, including the genera *Cotylogaster* Monticelli, 1892, *Cotylaspis* Leidy, 1857 (type genus) and *Lissemysia* Sinha, 1935 [14], are characterised by a ventral disc bearing three longitudinal rows of alveoli, whereas members of Aspidogastrinae, such as *Multicotyle* Dawes, 1941, *Lobatostoma*, *Aspidogaster* von Baer, 1826, *Lophotaspis* Looss, 1901, *Sychnocotyle* Ferguson, Cribb & Smales, 1999 and *Neosychnocotyle* Snyder & Tkach, 2007 [35], typically possess four longitudinal rows of alveoli [5]. Thus, the number of alveolar rows has traditionally been used as a key diagnostic character to differentiate subfamilies within Aspidogastridae. However, our phylogenetic analyses do not support this subfamilial classification, as both Aspidogastrinae and Cotylaspidinae were recovered as non-monophyletic. These results suggest that the number of alveolar rows, although taxonomically convenient, may not represent a reliable character for defining evolutionary lineages within the family.

## 5. Conclusions

This study provides the first integrative characterisation of *L*. *anisotremum*, refining its morphological and ultrastructural diagnosis and generating new 18S and 28S rDNA sequences. Phylogenetic analyses consistently recover *L*. *anisotremum* as sister to *L*. *manteri*, supported by both morphological similarities and their shared Pacific distribution. In contrast, the distinct phylogenetic position of *L*. *kemostoma* supports the non-monophyly of *Lobatostoma*, highlighting limitations of traditional diagnostic characters.

At higher taxonomic levels, the nested position of Multicalycidae within Aspidogastridae and the non-monophyly in traditionally defined subfamilies might suggest that characters such as the number of alveolar rows are not reliable indicators of evolutionary relationships. Although these findings could point to systematic incongruences, no taxonomic changes are proposed pending broader taxon sampling and comprehensive morphological reassessment. Overall, this study highlights the potential value of integrative taxonomy for exploring evolutionary relationships within Aspidogastrea. 

## Figures and Tables

**Table 1:** Information on Aspidogastrida species/lineages and outgroup sequences used to construct the 18S rDNA and 28S rDNA phylogenetic analysis (new sequences in bold).

Species	Family	18S	28S	Reference
*Aspidogaster conchicola*	Aspidogastridae	AJ287478	-	[27]
*Aspidogaster conchicola*	Aspidogastridae	DQ482608		[28]
*Aspidogaster conchicola*	Aspidogastridae		AY222162	[6]
*Aspidogaster ijimai*	Aspidogastridae	DQ482609		[28]
*Aspidogaster ijimai*	Aspidogastridae		MK387331-33	[25]
*Aspidogaster* sp.	Aspidogastridae		OR592551	[29]
*Cotylaspis* sp.	Aspidogastridae	AY222083	AY222165	[6]
*Cotylaspis* sp.	Aspidogastridae		KM457145	[30]
*Cotylogaster basiri*	Aspidogastridae	AY222082	AY222164	[6]
***Lobatostoma anisotremum***	**Aspidogastridae**	**PZ297966**	**PZ297967**	**Present study**
*Lobatostoma kemostoma*	Aspidogastridae		KF561238-39	[11]
*Lobatostoma manteri*	Aspidogastridae	L16911		[31]
*Lobatostoma manteri*	Aspidogastridae		AY157177	[32]
*Multicalyx cristata*	Multicalycidae		AY222163	[33]
*Multicalyx elegans*	Multicalycidae	AJ287532		[27]
*Multicalyx elegans*	Multicalycidae	DQ482610		[28]
*Multicotyle purvisi*	Aspidogastridae	AJ228785	AJ243684	[34]
*Multicotyle purvisi*	Aspidogastridae		AF023115	[35]
*Multicotyle purvisi*	Aspidogastridae		AY222166	[6]
*Neosychnocotyle maggiae*	Aspidogastridae		EF015578	[36]
*Polystomoides oris* *	Polystomatidae	FM992698		[37]
*Polystomoides siebenrockiella* *	Polystomatidae		FM992706	[36]
*Rugogaster hydrolagi*	Rugogastridae		AY157176	[32]
*Sychnocotyle kholo*	Aspidogastridae		EF015579	[36]
*Sychnocotyle* sp.	Aspidogastridae		EF015580	[37]

* Outgroup.

## Data Availability

The obtained sequences are deposited in GenBank under the accession numbers PZ297966, PZ297967.
